# Social deprivation as a risk factor for COVID-19 mortality among women and men in the UK Biobank: nature of risk and context suggests that social interventions are essential to mitigate the effects of future pandemics

**DOI:** 10.1136/jech-2020-215810

**Published:** 2021-04-27

**Authors:** Mark Woodward, Sanne A E Peters, Katie Harris

**Affiliations:** 1 The George Institute for Global Health, University of New South Wales, Sydney, New South Wales, Australia; 2 The George Institute for Global Health, School of Public Health, Imperial College London, London, UK; 3 Julius Center for Health Sciences and Primary Care, Utrecht, The Netherlands

**Keywords:** social class, infection, cardiovascular disease

## Abstract

**Objectives:**

To investigate sex differences in the effects of social deprivation on COVID-19 mortality and to place these effects in context with other diseases.

**Design:**

Prospective population-based study.

**Setting:**

UK Biobank.

**Participants:**

501 865 participants (54% women).

**Main outcome measure:**

COVID-19 as the underlying cause of death.

**Results:**

Of 472 946 participants alive when COVID-19 was first apparent in the UK (taken as 1 February 2020), 217 (34% women) died from COVID-19 over the next 10 months, resulting in an incidence, per 100 000 person years, of 100.65 (95% CI 79.47 to 121.84) for women and 228.59 (95% CI 194.88 to 262.30) for men. Greater social deprivation, quantified using the Townsend Deprivation Score, was associated with greater risk of fatal COVD-19. Adjusted for age and ethnicity, HRs for women and men, comparing those in the most with the least deprived national fifths, were 3.66 (2.82 to 4.75) for women and 3.00 (2.46 to 3.66) for men. Adjustments for key baseline lifestyle factors attenuated these HRs to 2.20 (1.63 to 2.96) and 2.62 (2.12 to 3.24), respectively. There was evidence of a log-linear trend in the deprivation–fatal COVID-19 association, of similar magnitude to the equivalent trends for the associations between deprivation and fatal influenza or pneumonia and fatal cardiovascular disease. For all three causes of death, there was no evidence of a sex difference in the associations.

**Conclusions:**

Higher social deprivation is a risk factor for death from COVID-19 on a continuous scale, with two to three times the risk in the most disadvantaged 20% compared with the least. Similarities between the social gradients in COVID-19, influenza/pneumonia and cardiovascular disease mortality, the lack of sex differences in these effects, and the partial mediation of lifestyle factors suggest that better social policies are crucial to alleviate the general medical burden, including from the current, and potential future, viral pandemics.

## Introduction

In the UK, the association between high levels of social deprivation and communicable diseases has long been recognised. The emergence of scientific^1 2^ and anecdotal evidence of higher rates of COVID-19 among the more deprived is then hardly a surprise. The link between social deprivation and non-communicable diseases was slower to emerge, but well established by the time of the seminal Black Report of 1980.^3^ This report showed that, despite the hopes of the welfare revolution that saw foundation of the National Health Service (NHS) in 1948, social gradients existed in total mortality rates and by multiple causes of disease, in young and old, in both sexes. More recent evidence^4 5^ has suggested that such social gradients have, if anything, increased.

Although the role of social deprivation in the emergent COVID-19 disease is now clear,^1 2^ whether the effects are similar to those for the leading causes of death among similar, common infectious diseases has yet to be established, which is important to ascertain given the probability of future novel viruses. Furthermore, given that current advice for healthy living tends to concentrate on lifestyle changes, it is useful to understand whether risks for COVID-19 are similar to those for cardiovascular disease (CVD), the leading cause of death among the class of non-communicable diseases. Most would agree that higher social deprivation tends to correlate with a greater chance of incident CVD, even if there is still a residual effect of social deprivation on CVD over and above lifestyle factors, such as smoking.^6 7^ Although a previous study has demonstrated the adverse effects of ‘unhealthy’ lifestyle factors on COVID-19 hospitalisation,^8^ the relative effects of such mediators of social deprivation in COVID-19 and common infectious and non-communicable diseases have yet to be explored.

We set out to resolve the above issues in a large cohort study, which offers greater flexibility, for example in controlling for confounders, and less chance of bias, most importantly that of indication bias,^9^ than when routinely collected data have been used, which is generally the case for COVID-19.^1 2^

## Methods

The UK Biobank is a population-based prospective cohort study that recruited over 500 000 individuals, with baseline data collected between 2006 and 2010.^10^ Individuals aged 40–69 were invited to attend one of the 22 centres for baseline assessment, which included questionnaires on a range of lifestyle and medical history, and physical and functional measurements. Written informed consent was obtained electronically for all participants.

### Baseline characteristics

The Townsend Deprivation Score is an area-based score of social deprivation (accounting for unemployment, overcrowding, non-car ownership and non-home ownership) that was determined immediately prior to the participant joining the Biobank, based on data from the preceding national census. Each participant was assigned a score corresponding to their postcode area. These areas have an average population of 309 individuals (approximately 125 households). The Townsend Deprivation Score was then grouped into equal fifths, such that the lowest fifth contained the 20% least socially deprived (least disadvantaged) and the highest fifth contained the 20% most deprived (most disadvantaged).^11^

Ethnic background was collected on participants, via touch screen, at a UK Biobank assessment centre. We categorised this as white (British, Irish, any other white background) or other (mixed, Asian or Asian British, Black or Black British, Chinese, other ethnic group; grouped together to avoid small numbers). Smoking status was self-reported as never, former or current smokers. Diabetes was also self-reported, and if the stated age at diagnosis was less than 30 and insulin use was reported the participant was classified as having type 1 diabetes, otherwise as type 2 diabetes. Medical history of CVD (myocardial infarction, stroke or angina) was also self-reported.

Body mass index (BMI) was calculated as the weight of the individual in kilograms, measured using the Tanita BC-418 MA body composition analyser, divided by the square of the individual’s standing height in metres. Blood pressure was taken at baseline using the Omron HEM-7015IT digital blood pressure monitor, by taking the mean of two sitting measures. Total cholesterol was measured using the Beckman Coulter AU580.

Further details on how variables were defined are provided in the online supplemental material.

10.1136/jech-2020-215810.supp1 Supplementary data

### Outcomes

Our primary outcome was death from COVID-19. The secondary outcomes were death from influenza or pneumonia and death from coronary heart disease or stroke (combined as CVD). Causes of death were obtained from NHS Digital for England and Wales and the NHS Central Register for Scotland. Deaths from COVID-19 were identified using emergency International Classification of Diseases-10 (ICD-10) codes based on definitions from the WHO^12^: (1) ‘U07.1 COVID-19, virus identified’, which was assigned to a disease diagnosis of COVID-19 confirmed by laboratory testing; and (2) ‘U07.2 COVID-19, virus not identified’, which was assigned to a clinical or epidemiological diagnosis of COVID-19 where laboratory confirmation was inconclusive or not available. Deaths from influenza or pneumonia were identified using ICD-10 codes J09-J18. Deaths from CVD were identified using ICD-10 codes I20, I21, I24, I25, 160, I61, I63 and I64. Follow-up was completed on 30 November 2020.

### Statistical methods

Baseline characteristics are presented as number (percentage) for categorical variables and as mean (SD) for continuous variables. Rates of death from the three underlying causes were estimated from Poisson models^13^ for the period from 1 February 2020 (taken as indicative of the start of COVID-19 in the UK) and across the entire follow-up for influenza/pneumonia and CVD. Cox proportional hazards models were used to quantify the association between fifths of Townsend score and death from COVID-19, influenza or pneumonia, and CVD from baseline to end of study follow-up. The least disadvantaged fifth was the reference category. Two sets of adjustments were employed: (1) for age and ethnicity; and (2) for age, ethnicity, baseline systolic blood pressure (SBP), diabetes, smoking, BMI, total cholesterol and CVD (multiple adjustment). We considered (1) to constitute our primary analysis, since age and ethnicity, unlike the other covariates, cannot be considered a consequence of social position. For both models, an interaction of sex with each variable was included to estimate HRs with 95% CIs to be extracted for women and men, as well as a relative comparison of HRs, as the women to men ratio of HRs (RHR).^13^ Penalised smoothing splines were used to examine the shape of the associations between continuous Townsend score and the study outcomes, by sex. If a log-linear relationship between Townsend score and risk of outcomes was deemed appropriate, Cox models with a continuous effect of Townsend score (per one-unit higher) were fitted. A sensitivity analysis was undertaken where any deaths that occurred prior to 1 February 2020 were excluded in the log-linear association analyses.

Predefined subgroup analyses were undertaken for the association of Townsend score and death from COVID-19, influenza or pneumonia, and CVD within subgroups, by sex, using Cox models. A priori, subgroups were defined by age (<60 years, ≥60 years), ethnicity (white, other), smoking (never, previous, current), diabetes (none, type 1 or type 2) and BMI (normal <25 kg/m², overweight from ≥25 kg/m² to <30 kg/m², obese ≥30 kg/m²) and baseline CVD (yes, no). P values for heterogeneity (for two-category subgroups) and for trend (for three-category ordinal subgroups) were recorded by sex and for the three-way interactions adding sex.

All analyses were undertaken using R V.4.0.2 (R Core Team, 2020).

## Results

Of the 501 865 participants in the UK Biobank that had Townsend score recorded (624 had missing scores), 54% were women. For both sexes, the mean age at baseline was 56 years and 94% were of white ethnicity (table 1). More men than women had history of CVD, had ever smoked and had diabetes at baseline; men had higher mean SBP. The least deprived 20% (nationally) made up more than 2.5 times as many as the 20% most deprived in the UK Biobank cohort, with similar ratios in women and men. The mean ages of both women and men decreased slightly with increasing social deprivation, from 57 years in the least disadvantaged fifth to 55 years in the most disadvantaged fifth (online supplemental table 1). The percentage of participants of non-white ethnicity, with CVD, with diabetes and who smoked all increased with increasing deprivation, as did the mean SBP and BMI. Mean cholesterol barely varied.

**Table 1:** Baseline characteristics of 501 865 UK Biobank participants, by sex

Characteristics	Women (n=273 048)	Men (n=228 817)
Age (years), mean (SD)	56.4 (8.0)	56.7 (8.2)
White ethnicity, n (%)	257 129 (94.2)	214 988 (94.0)
Townsend Deprivation Score		
Fifths, n (%)		
First; least disadvantaged	100 996 (37.0)	84 877 (37.1)
Second	56 590 (20.7)	46 179 (20.2)
Third	41 246 (15.1)	33 335 (14.6)
Fourth	36 909 (13.5)	30 456 (13.3)
Fifth; most disadvantaged	37 307 (13.7)	33 970 (14.8)
		
Systolic blood pressure (mm Hg), mean (SD)	135.3 (19.2)	140.9 (17.5)
Diabetes, n (%)		
No diabetes	261 403 (95.7)	211 320 (92.4)
Type 1	564 (0.2)	653 (0.3)
Type 2	9936 (3.6)	15 506 (6.8)
Smoking, n (%)		
Never	161 858 (59.3)	111 306 (48.6)
Former	85 349 (31.3)	87 508 (38.2)
Current	24 331 (8.9)	28 569 (12.5)
Body mass index (kg/m^2^), mean (SD)	27.1 (5.2)	27.8 (4.2)
Total cholesterol (mmol/L), mean (SD)	5.9 (1.1)	5.5 (1.1)
Previous cardiovascular disease, n (%)	10 093 (3.7)	20 434 (8.9)

During the median follow-up of 11.8 years, 577 (36% women) died of influenza or pneumonia and 4735 (26% women) died of CVD, with strong evidence of a systematic trend in increasing risk across increasing fifths of Townsend score for both causes of death (online supplemental table 2). There were 472 946 UK Biobank participants alive on 1 February 2020, the assumed start of the UK COVID-19 epidemic. By this time the mean age of survivors was 67.7 years in women and 67.9 years in men (online supplemental table 3). During 1 February–30 November 2020, 638 (34% women) died of COVID-19, compared with 55 (24% women) for pneumonia or influenza and 576 (31% women) for CVD (table 2). Age and ethnicity adjusted death rates tended to increase by increasing fifths of Townsend score for COVID-19, for both sexes, as they did for influenza/pneumonia and CVD.

**Table 2:** Age and ethnicity adjusted rates per 100 000 person years (with 95% CI) of COVID-19, influenza or pneumonia, and CVD, February–November 2020, by sex

Fifths	Women							Men						
	n	COVID-19		Influenza or pneumonia		CVD		n	COVID-19		Influenza or pneumonia		CVD	
		Deaths	Rate	Deaths	Rate	Deaths	Rate		Deaths	Rate	Deaths	Rate	Deaths	Rate
1	97 172	48	60.03 (37.33 to 82.73)	3	3.75 (0 to 9.43)	54	67.53 (41.33 to 93.73)	79 552	113	173.64 (128.25 to 219.03)	13	19.95 (5.44 to 34.45)	122	187.15 (137.68 to 236.62)
2	54 301	35	78.44 (40.04 to 116.85)	0	0 (NA)	40	89.64 (48.78 to 130.50)	42 969	78	219.10 (144.96 to 293.25)	9	25.62 (3.27 to 47.96)	87	247.85 (171.76 to 323.94)
3	39 513	28	86.30 (37.05 to 135.54)	3	9.21 (0 to 23.56)	30	92.41 (43.17 to 141.66)	30 907	55	218.11 (129.5 to 306.72)	2	7.89 (0 to 22.86)	54	213.48 (126.26 to 300.70)
4	35 232	47	159.41 (85.67 to 233.15)	5	17.37 (0 to 37.69)	25	86.70 (33.86 to 139.53)	27 910	68	290.44 (181.07 to 299.82)	8	35.14 (0 to 75.4)	52	224.36 (141.60 to 307.13)
5	35 160	59	205.92 (119.02 to 292.83)	2	6.95 (0 to 20.24)	29	101.05 (42.46 to 159.63)	30 230	107	414.69 (287.91 to 541.46)	10	40.89 (0.56 to 81.21)	83	331.62 (219.91 to 443.33)
Total	261 378	217	100.65 (79.47 to 121.84)	13	6.05 (1.61 to 10.5)	178	82.94 (64.54 to 101.34)	211 568	421	238.73 (203.59 to 273.87)	42	24.29 (12.92 to 35.66)	398	228.59 (194.88 to 262.30)

Negative confidence limits are expressed as zeros.

CVD, cardiovascular disease; NA, not applicable.

HRs by fifths of social deprivation, over the 11.8-year follow-up, showed these same trends after adjusting for SBP, diabetes, smoking, BMI, cholesterol and previous CVD, in addition to age and ethnicity (online supplemental table 4). Comparing the highest with the lowest fifth, the HR (95% CI) for COVID-19 death was 3.66 (2.82 to 4.75) for women and 3.00 (2.46 to 3.66) for men with age and ethnicity adjustment, and 2.20 (1.63 to 2.96) and 2.62 (2.12 to 3.24), respectively, after multiple adjustment. Broadly similar results were seen for both influenza/pneumonia and CVD, except that the HRs comparing the highest with the lowest fifths of Townsend score were higher in men than in women, with and without the multiple adjustment.

**Figure 1:**
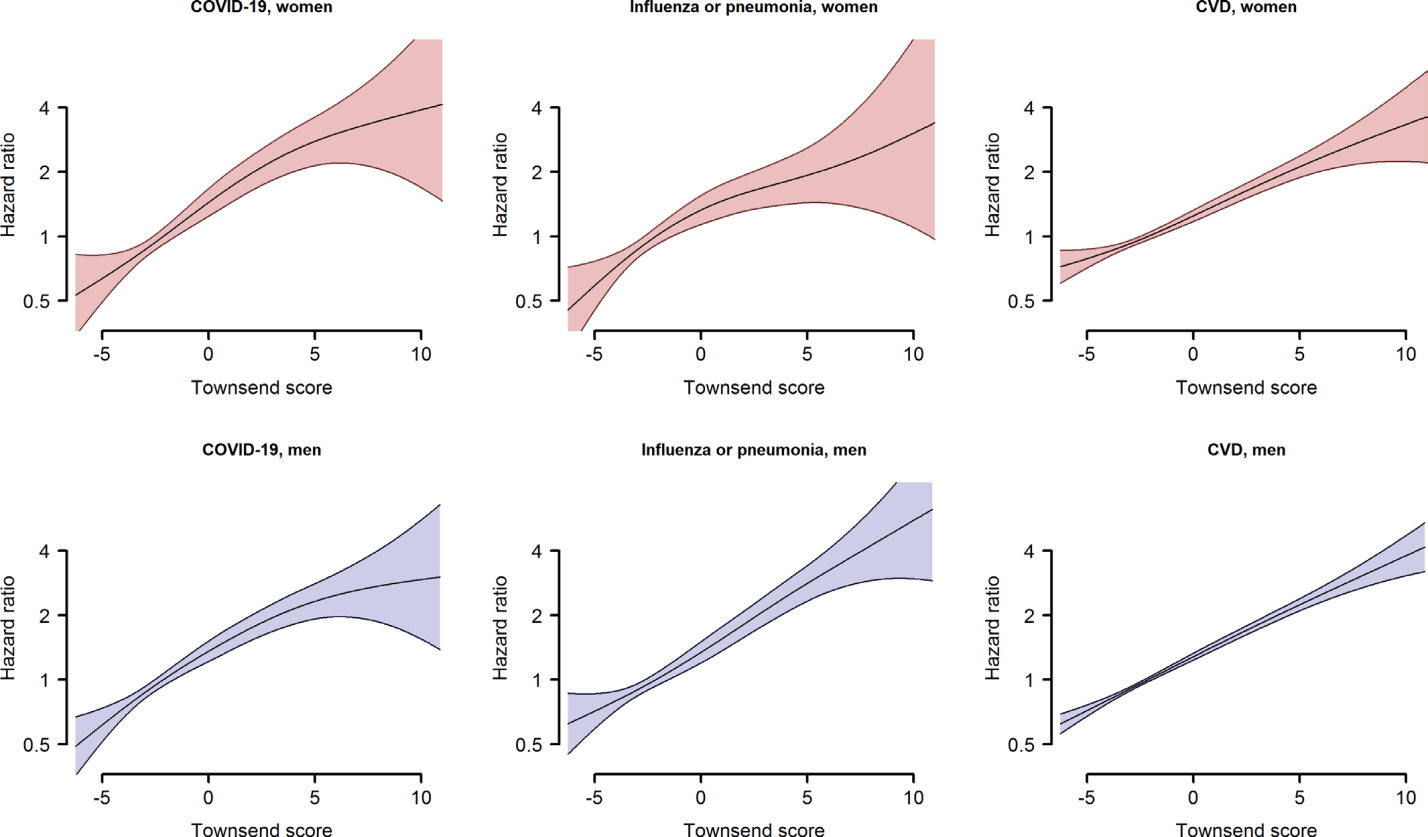
Penalised spline plots of age and ethnicity adjusted HR (with shaded 95% CI) for the association between Townsend score and death from COVID-19, influenza or pneumonia, and cardiovascular disease (CVD), by sex.

**Figure 2:**
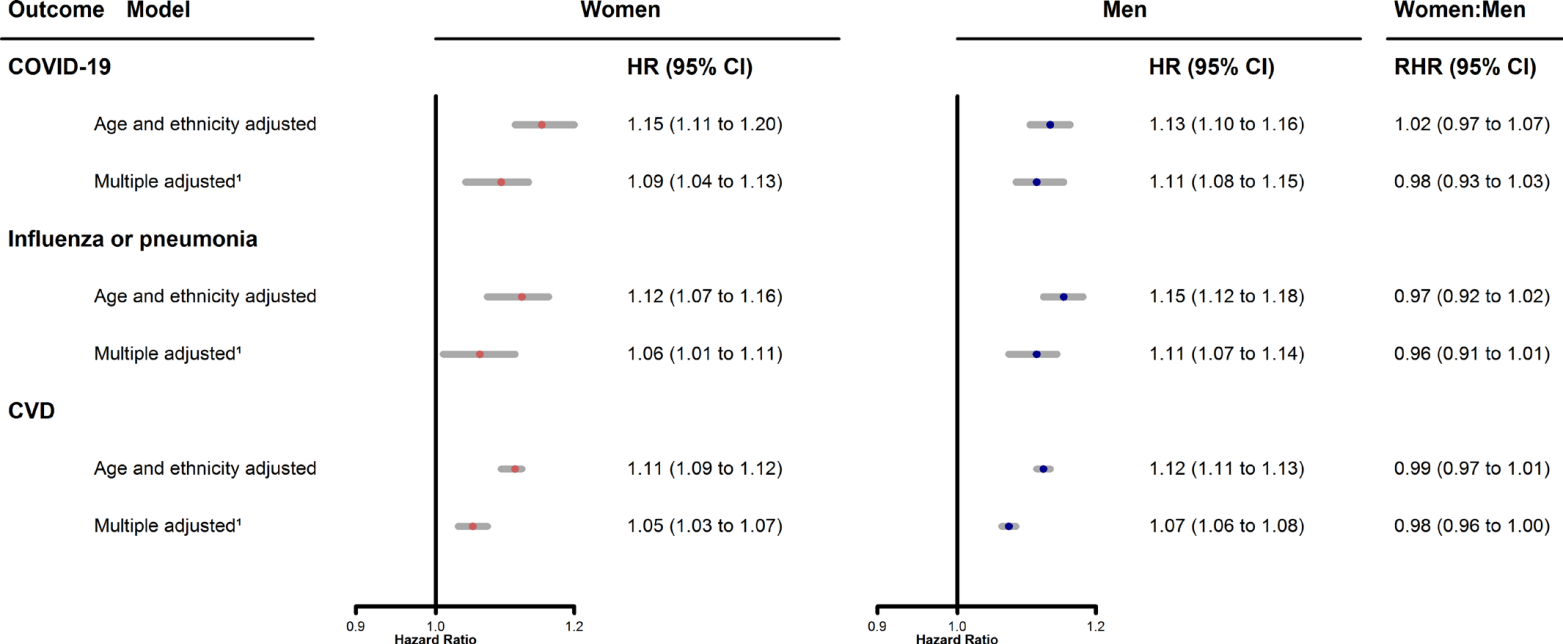
Age and ethnicity adjusted, and multiple adjusted^1^, HR (with 95% CI) for women and men, and women to men RHR (with 95% CI), for the association between one-unit higher Townsend score^2^ and death from COVID-19, influenza or pneumonia, and CVD.^1^Adjusted for baseline age, ethnicity, systolic blood pressure, diabetes, smoking, body mass index, total cholesterol and history of CVD. ^2^Townsend scores in the UK Biobank ranged from −6.26 to 11.00, with a median of −2.14. CVD, cardiovascular disease; RHR, ratio of HRs.

Spline curves demonstrated that the relationship between Townsend score and the HR for death from COVID-19 was approximately log-linear in age-adjusted and ethnicity-adjusted (figure 1) and multiple-adjusted (online supplemental figure 1) models. Where possible curvature was indicated it was in the extreme regions of the Townsend score, where precision of estimation is weak. Further, the shape and magnitude of the relationships between Townsend score and deaths from COVID-19, influenza or pneumonia, and CVD were all very similar for women and men. Hence a log-linear model seems appropriate, with results shown in figure 2. For fatal COVID-19, an increase of one unit in the Townsend score was associated with a 15% increase in risk in women (HR (95% CI): 1.15 (1.11 to 1.20)) and 13% in men (HR (95% CI): 1.13 (1.10 to 1.16)) after age and ethnicity adjustment, attenuating to 9% in women and 11% in men after multiple adjustment. The RHR was close to unity, with 95% CIs straddling 1, in both cases, suggesting no differential effect between the sexes. Similar results were found for influenza/pneumonia and CVD.

**Figure 3:**
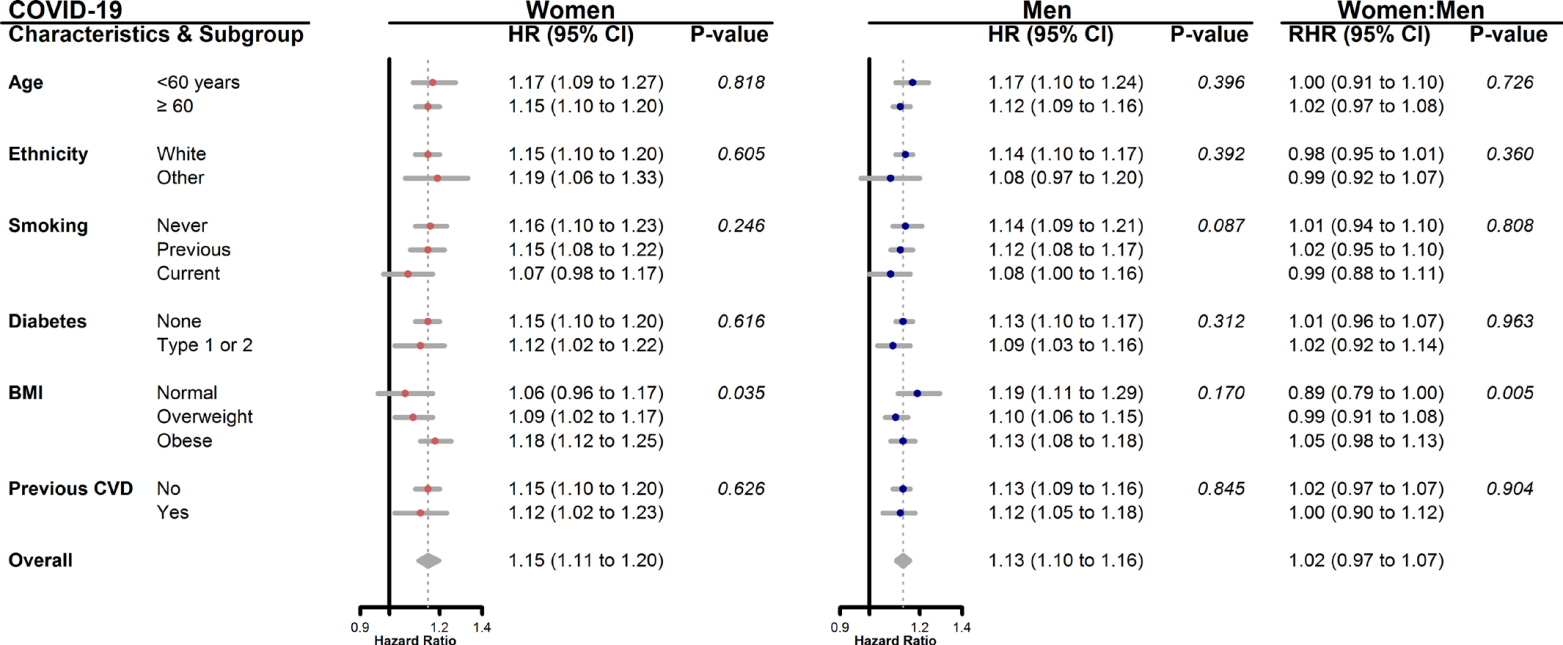
Age and ethnicity adjusted HR (95% CI) for women and men, and women to men RHR (with 95% CI), for the association between one-unit higher Townsend score and death from COVID-19, by subgroup.BMI, body mass index; CVD, cardiovascular disease; RHR, ratio of HRs.

Subgroup analyses by age, ethnicity, smoking, diabetes and previous CVD showed no evidence of a differential effect of deprivation on fatal COVID-19 for either women or men, nor between women and men (figure 3). However, deprivation was found to have little effect in women with BMI below 25 kg/m^2^, unlike in men for whom the effect was somewhat higher in those with normal weight than otherwise. Spline curves (online supplemental figure 2) showed a ‘J-shaped’ relationship between the HR for fatal COVID-19 and Townsend score only in women of normal BMI. For the secondary outcomes, the same conclusions were drawn for all but BMI. For fatal influenza/pneumonia, there was no evidence of interaction between deprivation and BMI for either sex, nor of a three-way interaction between deprivation, BMI and sex (online supplemental figure 3). For fatal CVD, the effect of deprivation was the same in women across all the three BMI groups considered, but in men the effect decreased with increasing BMI (online supplemental figure 4).

In the sensitivity analyses based only in the period from February to November 2020, HRs for influenza/pneumonia and CVD were similar to those observed in the main analysis across the entire follow-up (online supplemental table 5).

## Discussion

This analysis, of around half a million people in a general population, shows remarkably similar positive associations between the level of social disadvantage and the risk of death from COVID-19, influenza/pneumonia and CVD. In all cases, there was no evidence of an important sex difference in the effects of social deprivation, while accounting for the effects of lifestyle factors attenuated the effects, at most, only moderately.

Our results for influenza/pneumonia and CVD confirm what was expected from years of research work and government reports,^3–5^ and results for CVD events from a large linked study of general practice data in the UK,^7^ which used the Townsend score to quantify social deprivation, as here. For COVID-19, our results confirm the log-linear association of social deprivation found using linked routine UK data by Williamson *et al*,^1^ with a comparable index of social deprivation. Furthermore, the sex-specific effects on COVID-19 of the continuous Townsend social deprivation score found here are very similar to those reported using the same score in another linked UK routine database which analysed COVID-19 deaths and hospitalisations in the first half of 2020.^2^

To our knowledge, no previous study has compared the associations between social deprivation and COVID-19 and other causes of death using the same database, while relatively few studies quantify risk differences between the sexes in a direct way.^13^ Although our previous meta-analysis suggested that women may have a greater excess risk for CVD from lower social status than men,^14^ we did not find this for the Townsend score in an earlier analysis of myocardial infarction events in the UK Biobank population,^15^ with considerably shorter follow-up than in this report.

Although there is the expected social gradient in commonly accepted lifestyle risk factors, such as obesity and smoking, in the UK Biobank population, adjustment for these factors did not remove the association between social deprivation and risk across the three causes of death. We can only speculate that such issues as differential levels of education, low quality of housing and lack of finance at the lower end of the spectrum, and greater motivation and self-confidence at the upper end, are among several hypothetical explanations for the homogeneous social effect we have found.

The differential effect, by sex, of increasing deprivation on the risk of fatal COVID-19 according to BMI level seems to be due to a slight decrease in risk between those with very low to low deprivation only among women of normal BMI levels (below 25 kg/m^2^). Most likely this is a chance finding, unless reproduced in future research.

### Limitations

The UK Biobank cohort is comparatively socially advantaged and healthy, compared with the UK population in general, which may have biased our results, especially those on the absolute scale. Even if so, it is likely that any bias would be the same in both sexes. Non-white participants are poorly represented, which limits our scope for separating the effects of ethnicity and social deprivation. We restricted our analyses to underlying causes of death, and it is possible that different ways of defining cause would have produced different conclusions. Clearly there is a disconnect when using a cohort study to compare a disease that emerged a long time after study baseline with others that had immediate risk. We have chosen to use data from the complete follow-up in our main analyses, which gives the best power for the two secondary outcomes. We have implicitly assumed that social deprivation at baseline was the same when COVID-19 started to appear, around 11 years later, which is likely to be broadly true. If incorrect, our results will have underestimated the social gradients. Similarly, the lifestyle covariates at baseline are proxies for their 2020 levels, which compromises our secondary analyses of the effects of mediating variables, but not our primary (age and ethnicity adjusted) analyses.

## Conclusions

We have found that higher social deprivation is a risk factor for death from COVID-19, and from composite causes of death from both infectious diseases and non-communicable diseases, of a similar magnitude across the causes and between women and men. Taken with the residual effect, in each case, of social deprivation, after allowing for some of the lifestyle factors most commonly associated with low social status, we conclude that higher social deprivation is a fundamental harbinger of premature death. It is very reasonable to expect future virus outbreaks, and then, without prior widescale intervention, to experience the same social disparities in risk. Our data are from the UK, with its well-established single-payer healthcare system, which suggests that changes in social structure are required to address this modifiable risk factor.

**What is already known on this subject**

- Higher social deprivation is a risk factor for COVID-19.
- It is also a risk factor for major infectious diseases and cardiovascular disease.
- Several unhealthy lifestyle factors are more common in those of higher social deprivation.

**What this study adds**

- The relative risk for higher social deprivation, on a continuous scale, is similar for fatal COVID-19, fatal combined influenza and pneumonia, and fatal cardiovascular disease.
- In all cases these relative risks are much the same in women and men.
- Adjusting the relative risks for major lifestyle factors, at most, attenuates the effects without removing them.
- Improved social policies are needed to reduce health inequalities to mitigate against future pandemics, while also reducing the general health burden.

## Data Availability

Data are available upon reasonable request. Researchers can apply to use the UK Biobank resource and access the data used. No additional data are available.
