# Supplementary material for: Social deprivation as a risk factor for COVID-19 mortality among women and men in the UK Biobank: nature of risk and context suggests that social interventions are essential to mitigate the effects of future pandemics
Source: J Epidemiol Community Health. 2021 Apr 27:jech-2020-215810. doi: 10.1136/jech-2020-215810 (PMC8098299; doi:10.1136/jech-2020-215810)
Supplement: Supplementary file 1 [file jech-2020-215810supp001.pdf]

BMJ Publishing Group Limited (BMJ) disclaims all liability and responsibility arising from any reliance placed on this supplemental material which has been supplied by the author(s)

SUPPLEMENTARY MATERIAL

Supplementary table 1: Baseline characteristics of UK Biobank participants by fifths of Townsend score and sex

	Women					Men				
**Characteristic**	***1st* Least disadvantaged**	***2nd***	***3rd***	***4th***	***5th* Most disadvantaged**	***1st* Least disadvantaged**	***2nd***	***3rd***	***4th***	***5th* Most disadvantaged**
n	100996	56590	41246	36909	37307	84877	46179	33335	30456	33970
Age (years)	57.0 (7.8)	56.7 (7.9)	56.1 (8.1)	55.7 (8.2)	55.1 (8.3)	57.5 (7.9)	57.2 (8.1)	56.4 (8.3)	55.9 (8.4)	55.3 (8.4)
Ethnicity n (%)										
White	98545 (97.6)	54610 (96.5)	38859 (94.2)	33491 (90.7)	31624 (84.8)	82739 (97.5)	44556 (96.5)	31288 (93.9)	27597 (90.6)	28808 (84.8)
Other	2209 (2.2)	1767 (3.1)	2249 (5.5)	3205 (8.7)	5332 (14.3)	1784 (2.1)	1420 (3.1)	1858 (5.6)	2634 (8.6)	4734 (13.9)
Systolic Blood pressure (mmHg)	136 (19.3)	135.8 (19.2)	134.9 (19.1)	134.5 (19.2)	133.9 (19.2)	141.5 (17.2)	141.5 (17.4)	140.8 (17.4)	140.2 (17.6)	139.5 (18.0)
Diabetes n (%)										
No Diabetes	97978 (97.0)	54495 (96.3)	39520 (95.8)	34908 (94.6)	34504 (92.5)	79886 (94.1)	43116 (93.4)	30847 (92.5)	27658 (90.8)	29813 (87.8)
Type 1	163 (0.2)	115 (0.2)	89 (0.2)	92 (0.2)	105 (0.3)	206 (0.2)	116 (0.3)	81 (0.2)	116 (0.4)	134 (0.4)
Type 2	2615 (2.6)	1804 (3.2)	1487 (3.6)	1698 (4.6)	2332 (6.3)	4543 (5.4)	2790 (6.0)	2234 (6.7)	2441 (8.0)	3498 (10.3)
Smoking n (%)										
Never	65193 (64.6)	34506 (61.0)	23955 (58.1)	20036 (54.3)	18168 (48.7)	44960 (53.0)	23288 (50.4)	16078 (48.2)	13743 (45.1)	13238 (39.0)
Former	29983 (29.7)	17787 (31.4)	13361 (32.4)	12159 (32.9)	12059 (32.3)	32868 (38.7)	18041 (39.1)	12948 (38.8)	11500 (37.8)	12151 (35.8)
Current	5436 (5.4)	4039 (7.1)	3727 (9.0)	4463 (12.1)	6666 (17.9)	6725 (7.9)	4616 (10.0)	4109 (12.3)	4991 (16.4)	8128 (23.9)
BMI (kg/m^2)	26.6 (4.7)	26.9 (5.0)	27.1 (5.2)	27.6 (5.6)	28.3 (6.1)	27.6 (3.9)	27.8 (4.1)	27.9 (4.3)	28.0 (4.6)	28.2 (4.9)
Total cholesterol (mmol/L)	5.9 (1.1)	5.9 (1.1)	5.9 (1.1)	5.8 (1.1)	5.7 (1.1)	5.5 (1.1)	5.5 (1.1)	5.5 (1.1)	5.4 (1.1)	5.4 (1.2)
Previous CVD n (%)	2844 (2.8)	1863 (3.3)	1422 (3.4)	1619 (4.4)	2345 (6.3)	6482 (7.6)	3887 (8.4)	2899 (8.7)	2975 (9.8)	4191 (12.3)

Supplementary table 2: Age and ethnicity adjusted rates (per 100,000 person years) with 95% confidence intervals of influenza or pneumonia, and CVD from entry to the UK Biobank to 30 November 2020, by sex

	Women					Men				
		Influenza or pneumonia		CVD			Influenza or pneumonia		CVD	
**Fifth**	**n**	**Deaths**	**Rate**	**Deaths**	**Rate**	**n**	**Deaths**	**Rate**	**Deaths**	**Rate**
1	100,996	50	4.29 (2.56 to 6.03)	363	31.16 (26.58 to 35.74)	84,877	99	10.32 (7.43 to 13.21)	937	96.99 (87.81 to 106.17)
2	56,590	41	6.15 (3.60 to 8.70)	227	34.82 (28.08 to 41.56)	46,179	65	12.56 (7.96 to 17.16)	642	123.32 (109.06 to 137.59)
3	41,246	34	7.22 (3.50 to 10.94)	182	38.43 (30.08 to 46.79)	33,335	39	10.54 (5.68 to 15.40)	514	136.59 (118.61 to 154.87)
4	36,909	40	9.61 (5.65 to 13.56)	224	53.28 (42.65 to 63.92)	30,456	61	18.20 (11.01 to 25.39)	564	166.32 (145.05 to 187.60)
5	37,307	40	9.23 (4.61 to 13.85)	255	60.40 (48.76 to 72.04)	33,970	108	28.66 (20.36 to 36.97)	827	220.94 (197.45 to 244.00)
Total	273,048	205	6.49 (5.16 to 7.83)	1251	39.90 (36.57 to 43.23)	228,817	372	14.45 (12.23 to 16.66)	3484	134.80 (127.99 to 141.62)

Supplementary table 3: Baseline characteristics of 472, 946 UK Biobank participants alive on 1st February 2020, by sex

Characteristic	Women (n=261,378)	Men (n=211,568)
Age[a] (years), mean (sd)	67.7 (8.0)	67.9 (8.2)
White ethnicity, n (%)	246,113 (94.1)	198,665 (93.8)
Townsend deprivation score		
Fifths, n (%)		
1st Least disadvantaged	97,172 (37.2)	79,552 (37.6)
2nd	54,301 (20.8)	42,969 (20.3)
3rd	39,513 (15.1)	30,907 (14.6)
4th	35,232 (13.5)	27,910 (13.2)
5th Most disadvantaged	35,160 (13.5)	30,230 (14.3)
Continuous (per unit)	-1.35 (3.03)	-1.30 (3.12)
Systolic blood pressure (mmHg), mean (sd)	135.1 (19.1)	140.7 (17.3)
Diabetes, n (%)		
No Diabetes	250,879 (96.0)	196,925 (93.1)
Type 1	485 (0.2)	542 (0.3)
Type 2	8928 (3.4)	12,894 (6.1)
Smoking, n (%)		
Never	156,458 (59.9)	105,844 (50.0)
Former	81,191 (31.1)	79,585 (37.6)
Current	22,332 (8.5)	24,857 (11.8)
Body mass index (kg/m^2), mean (sd)	27.1 (5.2)	27.8 (4.2)
Total cholesterol (mmol/L), mean (sd)	5.9 (1.1)	5.5 (1.1)
Previous CVD, n (%)	8900 (3.4)	16,770 (7.9)

[a] Age of participants at 1st February 2020

Supplementary table 4: Age and ethnicity adjusted, and multiple adjusted[1] hazard ratios (HRs, with 95% confidence intervals(CIs)), and women to men ratio of hazard ratios (RHRs, with 95% CIs) for the association between fifths of Townsend score and death from COVID-19, influenza or pneumonia, and cardiovascular disease (CVD) by sex.

	Age and ethnicity adjusted			Multiple adjusted[1]		
**Townsend score**	**Women HR (95%CI)**	**Men HR (95%CI)**	**RHR (95% CI)**	**Women HR (95%CI)**	**Men HR (95%CI)**	**RHR (95% CI)**
COVID-19						
1st Least disadvantaged	Ref	Ref	Ref	Ref	Ref	Ref
2nd	1.34 (0.96 to 1.87)	1.32 (1.06 to 1.66)	1.01 (0.68 to 1.51)	1.37 (0.99 to 1.92)	1.31 (1.03 to 1.66)	1.05 (0.70 to 1.58)
3rd	1.55 (1.07 to 2.24)	1.45 (1.12 to 1.89)	1.07 (0.68 to 1.68)	1.33 (0.90 to 1.96)	1.46 (1.11 to 1.91)	0.91 (0.57 to 1.47)
4th	2.98 (2.23 to 3.98)	2.10 (1.65 to 2.67)	1.42 (0.97 to 2.07)	2.21 (1.62 to 3.00)	2.00 (1.56 to 2.56)	1.11 (0.74 to 1.64)
5th Most disadvantaged	3.66 (2.82 to 4.75)	3.00 (2.46 to 3.66)	1.22 (0.88 to 1.69)	2.20 (1.63 to 2.96)	2.62 (2.12 to 3.24)	0.84 (0.58 to 1.21)
Influenza or pneumonia						
1st Least disadvantaged	Ref	Ref	Ref	Ref	Ref	Ref
2nd	1.46 (1.07 to 1.99)	1.25 (0.98 to 1.59)	1.17 (0.79 to 1.74)	1.27 (0.92 to 1.76)	1.20 (0.93 to 1.55)	1.06 (0.70 to 1.60)
3rd	1.82 (1.30 to 2.55)	1.13 (0.83 to 1.55)	1.61 (1.02 to 2.55)	1.52 (1.07 to 2.16)	1.02 (0.73 to 1.42)	1.49 (0.92 to 2.41)
4th	2.52 (1.85 to 3.44)	2.06 (1.60 to 2.64)	1.23 (0.82 to 1.83)	1.98 (1.44 to 2.72)	1.71 (1.31 to 2.24)	1.16 (0.76 to 1.75)
5th Most disadvantaged	2.60 (1.90 to 3.57)	3.47 (2.86 to 4.21)	0.75 (0.52 to 1.09)	1.56 (1.09 to 2.21)	2.56 (2.06 to 3.18)	0.61 (0.40 to 0.92)
CVD						
1st Least disadvantaged	Ref	Ref	Ref	Ref	Ref	Ref
2nd	1.14 (1.00 to 1.30)	1.30 (1.20 to 1.41)	0.87 (0.75 to 1.02)	1.01 (0.88 to 1.16)	1.20 (1.11 to 1.31)	0.84 (0.71 to 0.98)
3rd	1.33 (1.15 to 1.54)	1.54 (1.41 to 1.68)	0.86 (0.73 to 1.02)	1.22 (1.05 to 1.42)	1.35 (1.23 to 1.48)	0.90 (0.76 to 1.07)
4th	1.92 (1.68 to 2.19)	1.96 (1.80 to 2.12)	0.98 (0.84 to 1.15)	1.48 (1.29 to 1.70)	1.57 (1.43 to 1.71)	0.94 (0.80 to 1.11)
5th Most disadvantaged	2.30 (2.03 to 2.60)	2.75 (2.56 to 2.95)	0.84 (0.72 to 0.96)	1.43 (1.24 to 1.64)	1.83 (1.69 to 1.97)	0.78 (0.67 to 0.92)

[1] Adjusted for age, ethnicity, systolic blood pressure, diabetes, smoking, BMI, total cholesterol, and previous CVD.

Supplementary table 5: Age and ethnicity adjusted hazard ratios (HRs, with 95% confidence intervals (CIs)) by sex, and women to men ratio of hazard ratios (RHRs, with 95% CIs) for the association between one unit higher Townsend score and death from COVID-19, influenza or pneumonia, and cardiovascular disease (CVD) by sex, using data left censored at 1st February 2020.

Outcome	Women HR (95% CI)	Men HR (95% CI)	RHR (95% CI)
**COVID-19**	1.15 (1.11 to 1.20)	1.13 (1.10 to 1.16)	1.02 (0.97 to 1.07)
**Influenza or pneumonia**	1.16 (1.00 to 1.35)	1.10 (1.01 to 1.20)	1.05 (0.88 to 1.26)
**CVD**	1.06 (1.01 to 1.11)	1.09 (1.06 to 1.12)	0.97 (0.92 to 1.03)

Note that the COVID-19 results must be the same as when the entire UK Biobank follow-up is analysed

Supplementary figure 1: Penalised spline plots of multiple adjusted[1] hazard ratios (with shaded 95% confidence intervals) for the association between Townsend score and death from COVID-19, influenza or pneumonia, and cardiovascular disease (CVD), by sex

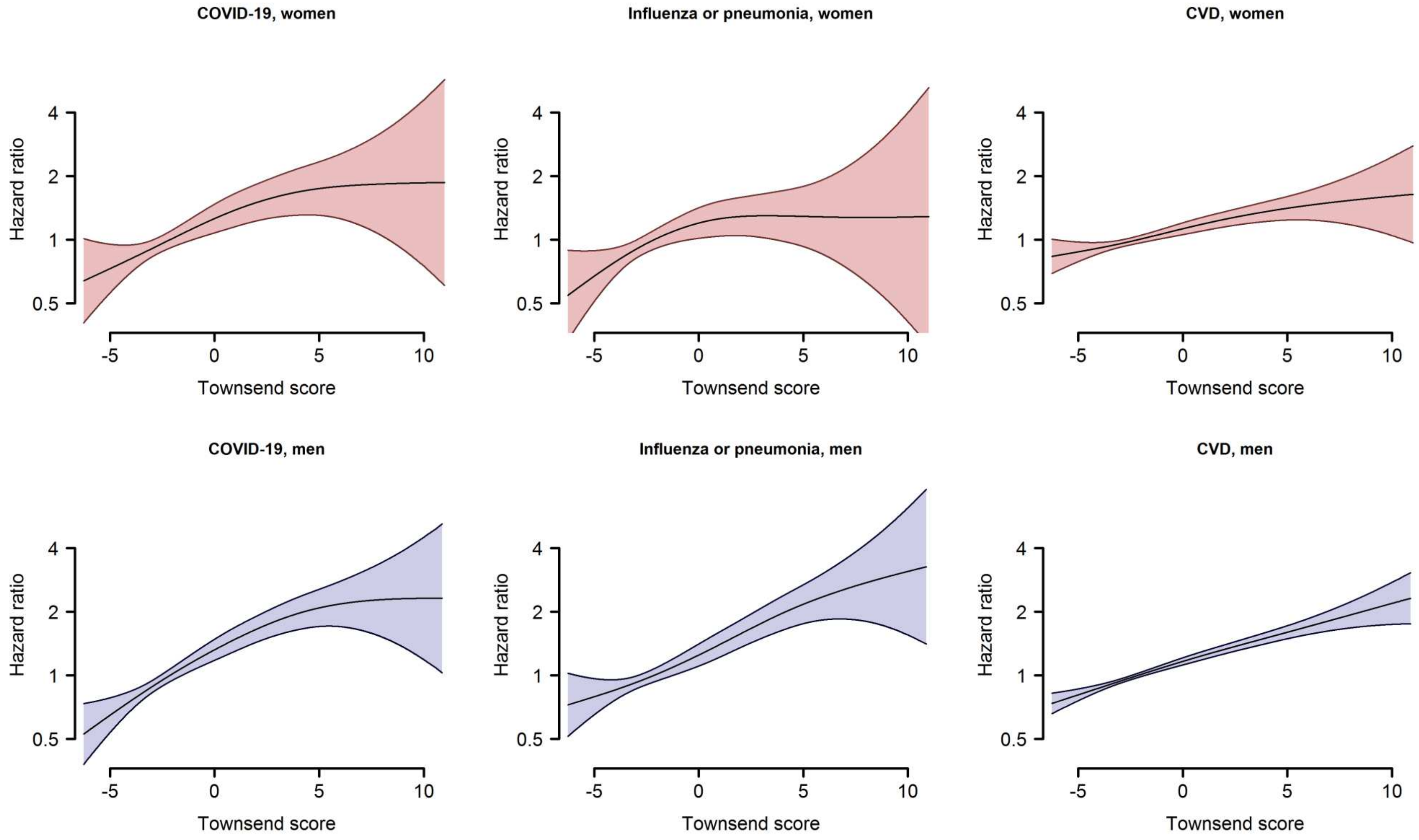

[1] Adjusted for age, ethnicity, systolic blood pressure, diabetes, smoking, BMI, total cholesterol, and previous CVD.

Supplementary figure 2: Penalised spline plots of age and ethnicity adjusted hazard ratios (with shaded 95% confidence intervals) for the association between Townsend score and death from COVID-19, by body mass index group. Red lines, and confidence intervals, for women; blue for men.

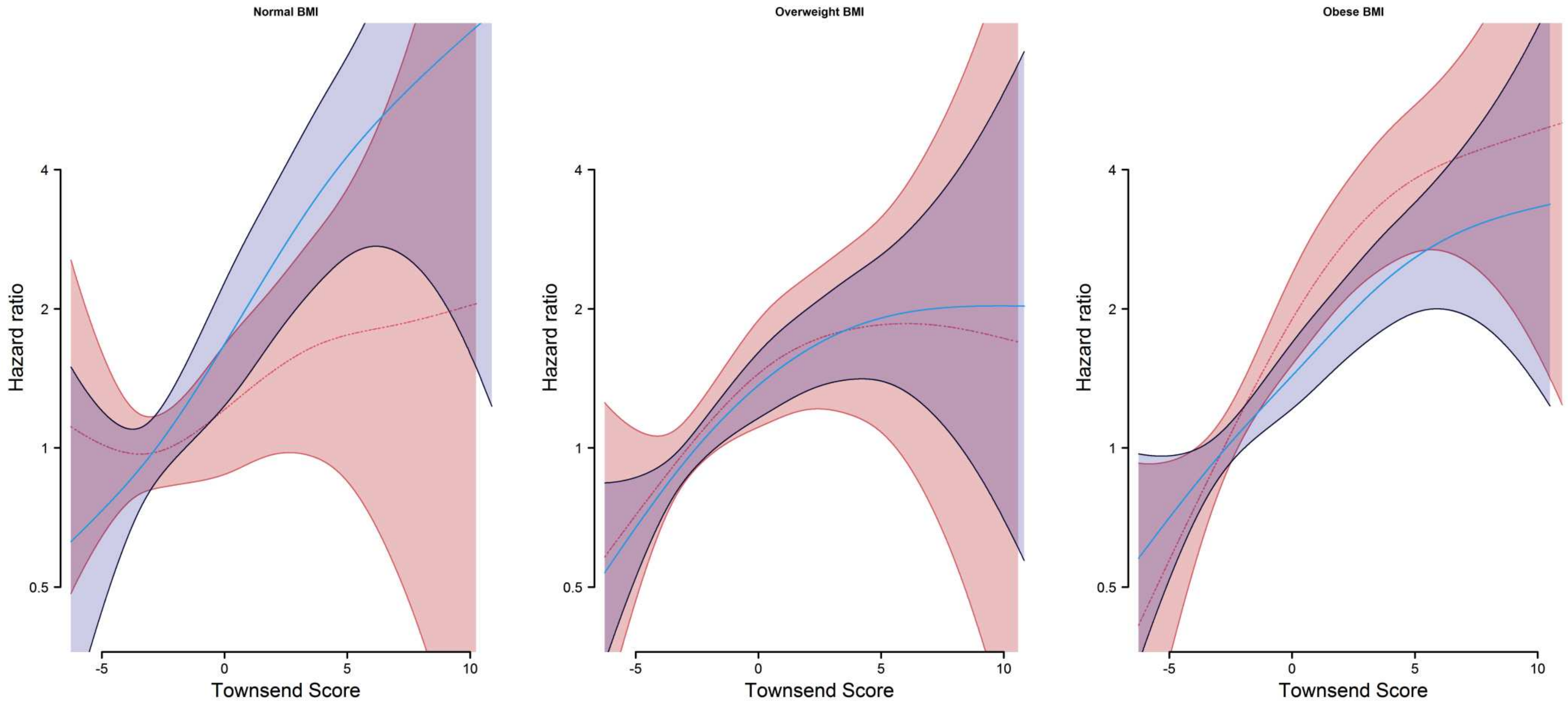

Supplementary figure 3: Age and ethnicity adjusted hazard ratios (HRs, with 95% confidence intervals (CIs)), and women-to-men ratio of HRs (RHRs, with 95%CIs), for the association between one unit higher Townsend score and death from influenza or pneumonia by sex and subgroup

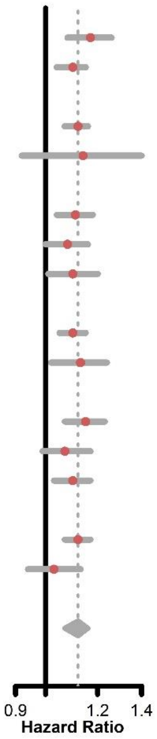

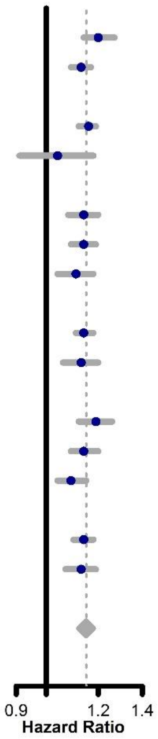

Influenza or pneumonia: Characteristics & Subgroup		Women: HR (95% CI)	Women: P-value	Men: HR (95% CI)	Men: P-value	Women:Men: RHR (95% CI)	Women:Men: P-value
**Age**	<60 years	1.17 (1.08 to 1.26)	*0.247*	1.20 (1.14 to 1.27)	*0.093*	0.97 (0.88 to 1.07)	*0.859*
	≥ 60	1.10 (1.04 to 1.15)		1.13 (1.09 to 1.17)		0.97 (0.92 to 1.03)	
**Ethnicity**	White	1.12 (1.07 to 1.16)	*0.864*	1.16 (1.12 to 1.19)	*0.116*	1.01 (0.98 to 1.04)	*0.337*
	Other	1.14 (0.92 to 1.40)		1.04 (0.91 to 1.18)		1.10 (0.95 to 1.27)	
**Smoking**	Never	1.11 (1.04 to 1.18)	*0.729*	1.14 (1.08 to 1.20)	*0.574*	0.97 (0.89 to 1.05)	*0.571*
	Previous	1.08 (1.00 to 1.16)		1.14 (1.09 to 1.19)		0.94 (0.87 to 1.03)	
	Current	1.10 (1.01 to 1.20)		1.11 (1.04 to 1.18)		0.99 (0.89 to 1.10)	
**Diabetes**	None	1.10 (1.05 to 1.15)	*0.697*	1.14 (1.11 to 1.18)	*0.668*	0.96 (0.91 to 1.02)	*0.406*
	Type 1 or 2	1.13 (1.02 to 1.24)		1.13 (1.06 to 1.20)		1.00 (0.89 to 1.12)	
**BMI**	Normal	1.15 (1.07 to 1.23)	*0.449*	1.19 (1.12 to 1.26)	*0.063*	0.96 (0.88 to 1.05)	*0.552*
	Overweight	1.07 (0.99 to 1.17)		1.14 (1.09 to 1.20)		0.94 (0.85 to 1.03)	
	Obese	1.10 (1.03 to 1.17)		1.09 (1.04 to 1.15)		1.01 (0.92 to 1.09)	
**Previous CVD**	No	1.12 (1.07 to 1.17)	*0.107*	1.14 (1.10 to 1.18)	*0.708*	0.98 (0.93 to 1.04)	*0.208*
	Yes	1.03 (0.94 to 1.13)		1.13 (1.07 to 1.19)		0.91 (0.82 to 1.02)	
**Overall**		1.12 (1.07 to 1.16)		1.15 (1.12 to 1.18)		0.97 (0.92 to 1.02)	

Supplementary figure 4: Age and ethnicity adjusted hazard ratios (HRs, with 95% confidence intervals (CIs)), and women-to-men ratio of HRs (RHRs, with 95%CIs), for the association between one unit higher Townsend score and death from cardiovascular disease (CVD) by sex and subgroup

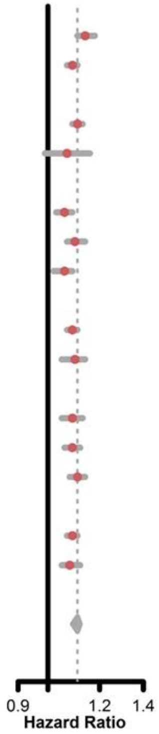

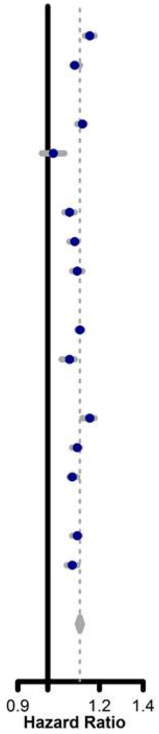

CVD Characteristics & Subgroup		Women HR (95% CI)	Women P-value	Men HR (95% CI)	Men P-value	Women:Men RHR (95% CI)	Women:Men P-value
**Age**	<60 years	1.14 (1.11 to 1.18)	*0.102*	1.16 (1.14 to 1.18)	*0.001*	0.99 (0.95 to 1.02)	*0.926*
	≥ 60	1.09 (1.07 to 1.11)		1.10 (1.09 to 1.12)		0.99 (0.97 to 1.01)	
**Ethnicity**	White	1.11 (1.09 to 1.13)	*0.449*	1.13 (1.11 to 1.14)	*< 0.001*	1.03 (1.02 to 1.04)	*0.115*
	Other	1.07 (0.99 to 1.16)		1.02 (0.98 to 1.06)		1.01 (0.97 to 1.05)	
**Smoking**	Never	1.06 (1.03 to 1.09)	*0.196*	1.08 (1.06 to 1.10)	*0.008*	0.98 (0.95 to 1.02)	*0.831*
	Previous	1.10 (1.07 to 1.14)		1.10 (1.08 to 1.11)		1.01 (0.98 to 1.04)	
	Current	1.06 (1.02 to 1.09)		1.11 (1.09 to 1.13)		0.95 (0.91 to 0.99)	
**Diabetes**	None	1.09 (1.07 to 1.11)	*0.912*	1.12 (1.11 to 1.13)	*0.001*	0.98 (0.96 to 1.00)	*0.088*
	Type 1 or 2	1.10 (1.05 to 1.14)		1.08 (1.05 to 1.10)		1.02 (0.97 to 1.06)	
**BMI**	Normal	1.09 (1.05 to 1.13)	*0.249*	1.16 (1.13 to 1.18)	*< 0.001*	0.94 (0.91 to 0.98)	*0.003*
	Overweight	1.09 (1.06 to 1.12)		1.11 (1.09 to 1.12)		0.99 (0.95 to 1.02)	
	Obese	1.11 (1.08 to 1.14)		1.09 (1.08 to 1.11)		1.02 (0.99 to 1.05)	
**Previous CVD**	No	1.09 (1.07 to 1.11)	*0.716*	1.11 (1.09 to 1.12)	*0.177*	0.99 (0.96 to 1.01)	*0.963*
	Yes	1.08 (1.05 to 1.12)		1.09 (1.07 to 1.11)		0.99 (0.96 to 1.03)	
**Overall**		1.11 (1.09 to 1.12)		1.12 (1.11 to 1.13)		0.99 (0.97 to 1.01)	

Supplementary methods – description of variable creation

Age – Provided by UK Biobank (Age when attended assessment centre).

Ethnicity – Participants were asked "what is your ethnic group?" during the initial Assessment Centre visit as part of the touchscreen questionnaire. At the top level the options were (White, Mixed, Asian or Asian British, Black or Black British, Chinese, Other Ethic Group, Do not know, prefer not to answer) an amalgam of sequential branching questions then garnered more details (i.e. if the participant answered White they were then asked (British, Irish, any other white background, prefer not to answer). The authors coded the following as White (British, Irish, any other white background, White) and the remaining options as other.

Townsend – Provided by UK Biobank (Townsend deprivation index calculated immediately prior to participant joining UK Biobank. Based on the preceding national census output areas. Each participant is assigned a score corresponding to the output area in which their postcode is located)

Fifths - Townsend Deprivation Score was then grouped into equal fifths by the authors (using the resource https://www.statistics.digitalresources.jisc.ac.uk/dataset/2011-uk-townsend-deprivation-scores), such that the lowest fifth contained the 20% least socially deprived (least disadvantaged), and the highest fifth contained the 20% most deprived (most disadvantaged)

SBP – Systolic Blood pressure provided by UK Biobank

1) (Blood pressure, automated reading, systolic. Two measures of blood pressure were taken a few moments apart. Range returned by the Omron device is 0-255)
2) (Blood pressure, manual reading, systolic. Two measures of blood pressure were taken a few moments apart. A manual sphygmometer was used if the standard automated device could not be employed).

The authors calculated the mean of the two baseline measures to represent baseline SBP.

Diabetes – Participants answered a question "Has a doctor ever told you that you have diabetes?", if the answer was affirmative, they were then asked "What was your age when the diabetes was first diagnosed? The authors derived type 1 and type 2 diabetes. If the stated age at diagnosis was less than 30 and insulin use was reported, the participant was classified as having type 1 diabetes; otherwise as type 2 diabetes.

Smoking – Derived by UK Biobank as Current, Previous, Never, prefer not to answer (using variables "Current tobacco smoking" and "Past tobacco smoking")

BMI – Provided by the UK Biobank (BMI value here is constructed from height and weight measured during the initial Assessment Centre visit. Value is not present if either of these readings were omitted)

Total cholesterol – Provided by the UK Biobank. Measured by CHO-POD analysis on a Beckman Coulter AU5800. Cholesterol was available from a range of key biochemistry markers that were measured in the blood sample collected at recruitment (for all 500,000 participants).

Previous CVD - Medical history of CVD was self-reported, based on a question asking the participant whether they had vascular/heart problems diagnosed by a doctor. The authors coded up a positive answer of MI, Stroke or Angina to indicate previous CVD.
